# Meniscal Tear Repair: What's New in the Literature?

**DOI:** 10.1155/tsm2/5511916

**Published:** 2025-10-04

**Authors:** Ahmad Hammad, Ahmad Naja, Mohamad Nassereddine

**Affiliations:** Department of Orthopedics Surgery, American University of Beirut, Beirut, Beirut Governate, Lebanon

**Keywords:** biological products, meniscal repair, meniscus, tibial meniscus injuries

## Abstract

Meniscal tear repair has become the gold standard modality for treating different types of meniscal tears. Despite the availability of numerous repair techniques, the ideal approach remains unclear, especially for complex and irreparable tears. Recently, innovative techniques have emerged to address these challenges, including hybrid/salvage techniques, meniscal scaffolds, and the introduction of biologics as part of the treatment. However, to date, no comprehensive review has provided an overview of the latter adjuncts to surgical intervention and aid in the management of irreparable meniscal tears. This study aims to fill this gap by examining the most common meniscal tear repair techniques, with a particular emphasis on the latest advancements in hybrid and salvage strategies. In addition, we will explore the role of biologics, including platelet-rich plasma and stem cells, in enhancing repair outcomes and assisting less experienced surgeons in treating complex, irreparable tears.

## 1. Introduction

Meniscal tears are a common knee injury, with an average annual incidence of 66 per 100,000 people [1]. Previously, it was believed that the menisci were cartilage structures in the knee joint with no functional purpose; hence, they were often completely removed through open total meniscectomy [1]. However, it was later discovered that insufficient removal of the meniscus could lead to the failure of meniscectomy. In 1948, Fairbank found that patients who underwent total meniscectomies experienced negative outcomes such as flattening of the condyle, narrowing of the joint space, and ridge formation [2].

Meniscal tears are common across all ages and impose a sizable annual cost burden of nearly $5 billion on the US healthcare system with over a quarter of patients unable to access care due to low income, young age, and other barriers to care [3]. In addition, in the past few decades, there has been a trend to preserve the meniscus, and surgeries were implemented to salvage it in aims to prevent early onset arthritis. Recent studies have shown that the function of the knee is directly related to the amount of residual meniscal tissue. As a result, there has been a shift towards preserving the meniscus whenever possible [4]. To optimize the outcomes of meniscal repair, several biologic therapies, including platelet-rich plasma (PRP) and stem cells have been incorporated as part of treatment protocols to enhance outcomes, healing, and functional recovery [5].

This review aims to explore the available evidence on meniscal tear management and the available conservative and surgical treatment options. In the light of the new modalities in treating meniscal tears, we will highlight the recent salvage techniques including the benefits of the use of biologics and PRP in meniscal tear treatment.

## 2. Materials and Methods

We conducted a quantitative synthesis of all studies comparing different surgical treatments of meniscal tears, according to the Preferred Reporting Items for Systematic Reviews and Meta-Analyses (PRISMA) guidelines with a PRISMA checklist and algorithm [6, 7]. A literature review was performed using Cochrane Library, PubMed, MEDLINE, and Scopus, and no restrictions were made regarding language, publication status, and clinical study design, and no date restriction was set [8]. This is a retrospective review article that did not require approval from the institutional review board.

The search strategy used the following medical subject headings (MeSH) and terms: Meniscal tear AND treatment, Meniscal tear AND surgery, Complex OR Salvage AND meniscal tear, Medial meniscal tear, Lateral meniscal tear, Biologics AND meniscal tear, platelet-rich plasm AND meniscal repair.

Supplemental data were identified through a random search on Google and Google Scholar. A search of the references of recent meta-analyses on the subject was also completed. For further information regarding current trials, the ClinicalTrials.gov [9] registry platform was searched using the following MeSH terms: meniscal tear and treatment.

**Figure 1:**
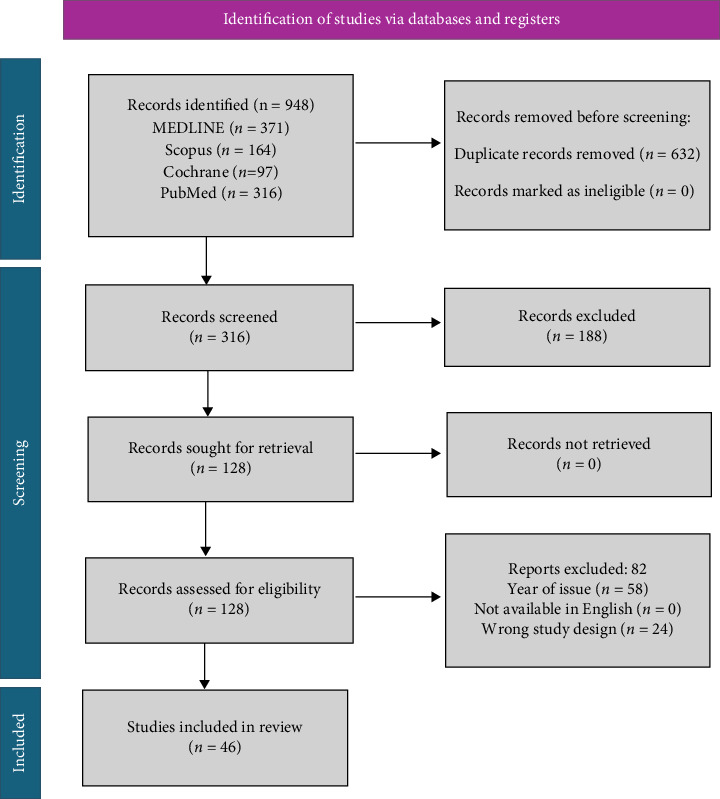
PRISMA flow diagram.

We systematically reviewed studies to determine if they met the inclusion criteria: English language studies, Level 1–4 studies, comparative and observational studies, and meniscal tear with no restriction to age (above 18) and sex. Exclusion criteria included case reports, case series, biomechanical studies, animal studies, review articles, articles not in the English language, and experts' opinions and reoperations. We limited our search to focus on more recent evidence on the latest treatment modalities in the last 10 years (between 2014 and 2024); however, only articles deemed of significance on the clinical practice were included in the study even if published before 2014. Initial search resulted in 948 records identified for screening; however, after duplicates and studies that did not meet the inclusion criteria as per study design and date of publication year were excluded, 128 articles were assessed, of which 46 were included. The search algorithm according to the PRISMA guidelines is shown in Figure 1.

### 2.1. Meniscal Tear Classification

**Figure 2:**
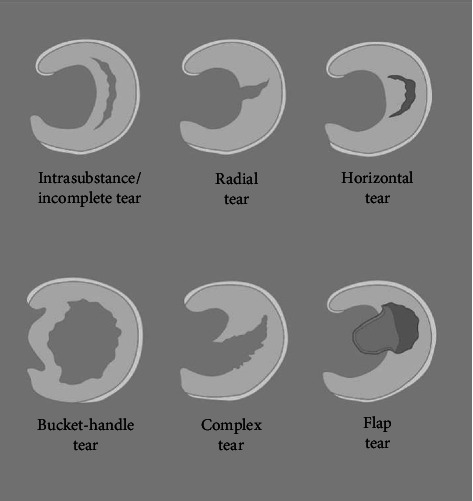
Schematic diagram of different meniscal tear subtypes.

Meniscal tears can be classified following the International Society of Arthroscopy, Knee Surgery and Orthopedic Sports Medicine (ISAKOS) system based on location (medial vs. lateral and red zone vs. white zone), pattern, and tear complexity (simple vs. complex vs. degenerative) [10]. According to orientation, tears are divided into incomplete intrasubstance, vertical radial, horizontal, oblique, bucket-handle, flap and complex tears as depicted in Figure 2 [11]. Longitudinal tears are more commonly found on the inner side of the knee (medially), while radial tears are more frequently observed on the outer side of the knee (laterally) [12]. This classification helps in understanding the specific characteristics and location of the tear.

Vertical longitudinal tears occur in the meniscus between the collagen fibers that run vertically [13]. These tears may not always disrupt the biomechanics of the knee and can be asymptomatic. However, complete vertical tears can twist within the joint, leading to “bucket-handle” tears, which are unstable and can cause mechanical symptoms or locking of the knee [14]. Vertical radial tears disrupt the collagen fibers that run circumferentially in the meniscus, affecting its ability to absorb the load between the tibia and the femur. These tears are usually not amenable to repair, and partial meniscectomy (removal of part of the meniscus) does not fully restore the function [15]. As a result, accelerated degenerative changes in the knee are likely to occur.

Horizontal tears split the meniscus into upper and lower parts and may exist without causing clinical symptoms [16]. They are usually mechanically stable but can lead to flap tears. The frequency of horizontal tears increases with age and is often accompanied by the development of meniscal cysts [17]. Oblique tears create flaps in the meniscus that are mechanically unstable and associated with mechanical symptoms [17]. Resection (removal) of the tear is necessary to prevent further propagation of the tear, as the flap can get caught within the joint, mainly during knee flexion [18]. Complex or degenerative tears refer to cases where two or more tear patterns exist. These tears are more common in the elderly and are often accompanied by osteoarthritic changes in the knee [19].

Seminal works by Arnoczky on meniscal biomechanics emphasize its vascularity, collagen structure, and mechanical behavior under load, highlighting the critical role of meniscal tissue in joint stability and load distribution, underscoring the importance of preserving meniscal integrity during surgical interventions to prevent long-term joint degeneration [20]. This landmark study by Arnoczky and Warren is one of the foundational works in meniscal biomechanics, particularly in understanding the vascular zones of the meniscus; it demonstrated that the outer one-third of the meniscus (the red zone) has a rich blood supply by genicular arteries, while the inner two-thirds (the white zone) are avascular. This distinction is important for surgical repair and healing, as the vascular zones play a key role in tissue regeneration. 10%–25% of the meniscal periphery is supplied by perimeniscal capillary plexus originating in the capsular and synovial tissues of the joint; also, the anterior and posterior horn attachments of the menisci have a good blood supply and are covered with vascular synovial tissue [20]. In addition, Fithian and Kelly explored the meniscus's crucial role in load distribution, shock absorption, and knee stability, and how these biomechanical roles are critical for knee joint health. He also discussed the advancements in meniscal repair techniques, emphasizing the need to preserve meniscal function to maintain optimal knee biomechanics [21].

### 2.2. Meniscal Tear Management

There has been a growing interest in avoiding meniscectomy and opting for meniscal repair. Animal studies conducted in the early 1980s showed that the meniscus has the ability to generate a healing response, particularly at its periphery [22]. A study by Cabaud et al. on transverse medial meniscal lacerations and repair with a single Dexon suture in canine and rhesus knee joints showed sufficient healing and protection of the underlying articular cartilage in 94% of the cases after just 4 months, with only 6% failing to heal [23]. Nonetheless, these results cannot be translated directly to humans due to the anatomical, structural, and biomechanical differences between human and animal joints and differences in joint load and function, in addition to variation in healing responses and regenerative capacities in terms of rapidity and scar tissue formation. Histology revealed that the scar tissue formed was composed of unorganized collagen without common ground substance components. These findings highlight the potential for meniscal repair as a viable alternative to meniscectomy, even in older adults.

Effective blood supply is pivotal for the successful outcome of meniscal repair procedures, especially tears situated in the red–red or potentially the red–white zone, where healing is anticipated [24]. However, the absence of blood vessels in the remaining meniscus restricts the widespread application of meniscal repair, often necessitating meniscectomy in patients. Efforts have been made to induce bleeding in avascular zones, such as utilizing exogenous fibrin clots to provoke a reparative response in these regions. In five cases of posterolateral meniscal tears anterior to the popliteus fossa, lacking penetrating blood vessels, repair supplemented with a fibrin clot demonstrated promising results, enabling all patients to return to their preinjury athletic levels, with second-look arthroscopy revealing peripheral healing in all instances. Moreover, trephination of vascular channels along the free meniscal edges has been shown to enhance healing rates [25]. A comparative study that combined meniscal repair with trephination exhibited a significantly lower retear rate compared to meniscal repair alone [26]. Additional evidence supporting the role of bleeding in aiding meniscal repair stems was observed in a study by Cannon and Vittori, who reported a 93% healing rate in patients undergoing meniscal repairs alongside anterior cruciate ligament (ACL) reconstruction, in contrast to 50% in those undergoing meniscal repair alone. ACL reconstruction entails tibial and femoral drilling, facilitating the delivery of local growth and clotting factors, potentially contributing to the elevated success rate of repairs. Furthermore, acute repairs within 8 weeks of injury demonstrated superior outcomes compared to more chronic repairs [27].

Although older adults may have less robust healing potential, the preservation of the meniscus through repair can improve long-term knee function, reduce the risk of osteoarthritis, and provide better overall joint health and functional mobility compared to meniscectomy by reducing the risk of osteoarthritis and preventing cartilage degeneration [28, 29]. Everhart et al. showed that meniscus repair involving adults aged 40 and older had success rates comparable to the younger population, and the rate of osteoarthritis was 53% in the meniscal repair group as compared to 99% in the meniscectomy group and 95% in the nonoperative group [28].

The inside-out technique is still considered the gold standard for meniscal repair by many surgeons. It is most suitable for tears of the posterior and middle thirds of the meniscus and allows more consistent suture placement perpendicular to the tear [30]. In this technique, incisions are made inside the knee for suture retrieval, after initial arthroscopic evaluation [15]. Sutures are introduced from inside the knee and knotted onto the capsule. Outside-in techniques, on the other hand, are more suitable for repair of the anterior and middle thirds of the meniscus [31]. Once the tear is identified through arthroscopy, the skin is transilluminated to locate the tear. A vertical mattress suture can then be used to repair the torn meniscus, aiming to reduce the risk of neurovascular injury [31].

Various all-inside arthroscopic meniscal repair devices have been developed. Earlier devices were rigid, while newer devices are suture-based [32]. The Meniscus Arrow was one of the first all-inside devices, consisting of a rigid, degradable polylactic acid arrow. Long-term studies showed a deterioration in the success rates of the rigid devices due to incomplete meniscal healing caused by degradation of the fixation device [33]. Rigid absorbable implants, such as the Mitek meniscus refixation device and Surgical Dynamics S.D. staple, were found to undergo hydrolysis, significantly reducing their failure strength over time [34]. Other rigid devices have also been introduced with similar issues, including complications such as chondral injuries, synovitis, implant migration, fragmentation, and soft tissue irritation. In view of complications, surgeons have abandoned the use of rigid devices. Suture-based implants have been developed to overcome the complications associated with rigid devices and allow for more controlled tensioning [35]. These implants consist of an anchor component and a sliding knot, which can compress the torn meniscal segments together. Examples include the FasT-Fix, which has shown early positive results in terms of Lysholm scores and failure rates [36]. More recently, and with the introduction of suture tapes to the market, the latter are found to have even superior biomechanical properties and better healing compared to conventional sutures [37]. Studies comparing rigid and suture devices found a lower failure rate (5%–10%) compared to rigid fixation (15%–20%), with a higher load to failure rate in suture implants (87.7 N vs. 56.3 N) [38]. In addition, rigid devices may be favored in patients over 40–50 years of age with acute tears due to their healing potential and faster recovery; however, younger active patients with traumatic complex tears are better treated with suture-based devices due to their flexibility but with longer rehabilitation [39]. While direct cost-effectiveness analyses for devices such as FasT-Fix and suture tapes are limited, existing research indicates that all-suture-based meniscal repair techniques are cost-effective due to the device's performance and its influence on improved clinical outcomes and, consequently, healthcare costs [40]. Sherman et al. found that meniscal repair using all-suture-based techniques decreases the cost by $12,227 per patient within a hospital setting and improves life quality [41]. Overall, while all-inside devices offer potential advantages, further research and improvements are needed to address complications, ensure the long-term success of these techniques, and assess the economic impact of these newer devices.

A study by Barber et al. on 41 meniscal repairs evaluated at follow-up of 30.7 months found a clinically effective meniscal repair in 83% of patients. Repeat arthroscopies were performed in 12 repairs, and failures were found in 7 (17%) cases. The most common adverse event encountered was toggling and pullout of the anchors during the insertion process [42]. Another study by Tachibana et al. on 46 patients undergoing 65 meniscal repairs at 14 months reported a clinical success rate of 83%, with 11 repairs failing and 9 showing incomplete healing during arthroscopy. There were six complications related to improper deployment, which required repeat procedures [43]. These studies highlight the importance of thorough evaluation and potentially a second-look arthroscopy to assess the success of meniscal repairs. Adverse events, such as anchor toggling and pullout, as well as improper deployment, can impact the outcome of the repair. Even though both all-inside and inside-out repairs demonstrate improved outcomes, a recent study by Dzidzishvili et al. suggests that the inside-out technique may have a higher failure rate clinically and on second-look arthroscopy [44]. Further research and advancements in techniques and devices are needed to address these complications and improve the success rates of all-inside meniscal repairs.

### 2.3. Biologics

The use of biologics to stimulate meniscal growth and enhance meniscal healing has been attempted through various techniques. The potential of enhancing the healing and regeneration of meniscal tissue following repair is inherently secondary to the growth factors and stem cells that promote growth and tissue integration [45]. Some approaches, such as injecting hyaluronic acid or growth factors, have not shown any improvement in meniscal healing or have produced inconsistent results [46]. On the other hand, using matrix metalloproteases that inhibit inflammatory factors in the meniscus has been successful in promoting meniscal healing. However, this approach has also led to musculoskeletal toxicity, and it did not provide clinical improvement in patients with arthritis [47]. Other techniques have shown promising results in promoting meniscal healing in preclinical trials but need further validation including the use of PRP, fibrin clots, stem cell therapy, and marrow venting [47]. Nonetheless, growth factors such as platelet-derived growth factor (PDGF), transforming growth factor–beta (TGF-β), and vascular endothelial growth factor (VEGF) help recruit and activate local stem cells and progenitor cells at cite of injury, thus stimulating cell growth, proliferation, differentiation, collagen synthesis, matrix remodeling, angiogenesis, and tissue repair [48, 49]. A recent systematic review by Utrilla et al. included 3 randomized controlled trials on the role of PRP in meniscal repair, and assessments combining MRI and arthroscopy indicated a significantly better failure rate in the PRP group but conflicting functional and pain results [50]. While various approaches are being explored, the use of biologics to enhance meniscal growth and healing is still an area that requires further research and validation to determine their efficacy and clinical significance. The challenges remain in protocol standardization, patient selection, cost, and accessibility.

### 2.4. Salvage Techniques

Meniscus reconstruction may be necessary when the remaining tissue is inadequate for proper fixation. A novel technique was proposed for meniscal repair in cases where traditional methods are not feasible. The technique involves creating circumferential-surrounding sutures from the joint capsule above to the capsule below the meniscus, encircling the perimeter of the meniscus, effectively “packing” and compressing the meniscal tear segments together. This method is particularly useful in cases with poor meniscal tissue quality or tear patterns that would usually be considered irreparable. Although this technique alters the normal shape of the meniscus free edge and requires a competent joint capsule, it provides a way to preserve the meniscus in scenarios where other options may not be possible [51]. It also offers promising potential for managing irreparable meniscal tears and preserving the function and integrity of the meniscus. While the success rates of this technique are yet to be identified, an all-inside, all-suture meniscus-to-meniscus circumferential compression stitch showed an 82.6% success rate for challenging horizontal cleavage tears at a 2-year follow-up [52].

In summary, rasp preparation of all tear surfaces, stable suture fixation, and injection of exogenous clot are the mainstay salvage methods for irreparable meniscal tears. For single longitudinal tears in chronic knees with peripheral white rims of 5 mm or greater, the use of a fascia sheath may improve the reliability of repair. The fascia sheath is particularly indicated for complex tears, including flaps and radial splits [53]. For irreparable bucket-handle chronic and displaced tears, a salvage technique was described in cases where reduction is not possible by anchoring the anterior end of the torn segment with an outside-in repair, or if the torn remnant is limited to the posterior half, an all-inside repair is possible [54].

Meniscal allograft transplantation is a treatment option for patients who have significant meniscal loss or damage, particularly those who are symptomatic and have a functional knee. However, it has restrictive indications and is not suitable for patients with instability, older age, axial malalignment, and diffuse subchondral bone exposure [55]. In some cases, correcting these conditions may make the patient eligible for a meniscal transplant. While improved clinical outcomes have been reported with allograft transplantation, there is limited high-quality literature with sufficient control groups. Several contraindications, however, should be considered and include patients less than 18 or more than 50–60 years of age, morbid obesity, severe knee osteoarthritis or inflammatory arthritis, ligamentous instability, and joint malalignment [56]. Nevertheless, transplantation remains one of the few options for meniscus-deficient knees to consider before resorting to arthroplasty [57].

To enhance the chances of meniscus healing, a structured rehabilitation program is deemed necessary. It is emphasized that tears located in the white substance of the meniscus are more sensitive to rapid return to weight-bearing compared to peripheral tears. Further research and clinical studies are needed to assess the efficacy and long-term outcomes of these novel repair techniques.

### 2.5. Meniscal Scaffolds

There has been extensive research on various meniscal prosthetics, with the most promising results seen in porous and stiffer scaffolds that allow for the growth of new cartilaginous tissue. The latter is achieved through the seeding of the scaffold with fibrochondrocytes, autologous chondrocytes, hyaluronic acid, or human connective tissue growth factors [58]. Other options include resorbable collagen meniscus implants made from bovine Achilles tendons, as well as improved meniscal scaffolds that fit through 3-dimensional printing [59]. Collagen scaffolds are biological implants derived from animal-derived or human collagen matrices, whereas synthetic scaffolds are made from various biomaterials (polyesters, polyurethane, or other synthetic polymers) to allow biocompatibility and mimic meniscus mechanical properties. Collagen scaffolds tend to have better long-term joint preservation and cartilage health, a more natural integration process, and lower complication risk of immune response or rejection but may have slower recovery times and potential for early failure. On the contrary, synthetic scaffolds offer immediate mechanical support and faster recovery times but may fall short in terms of long-term biological integration and durability. At 10 years follow-up, Reale et al. found no difference between both scaffold types in pain, function, and activity; significant improvement in International Knee Documentation Committee score and visual analog scale score; and 80% implant survival [60].

However, different meniscal replacements often encounter problems such as transplant tears, malpositioning, and misshaping and are generally inferior to native meniscal tissue in terms of tensile strength and compression. In addition, the clinical adoption of 3D-printed meniscal scaffolds is hindered by regulatory hurdles due to classification, material safety (e.g., biopolymers, hydrogels, and their adverse reactions in the body), surgical complexity, and the learning curve required for effective use and the insufficient long-term clinical data demonstrating sustained efficacy, safety, and long-term survival [61, 62]. Further comparative studies and advancements in methods are likely necessary before the adoption of these techniques.

### 2.6. PRP

Regenerative medicine, using stem cells and PRP, has been a breakthrough in medicine. The latter have been incorporated across different fields, including orthopedics and sports medicine. A recent meta-analysis by Sakti et al. found that meniscal repair when augmented with PRP results in a low failure rate, significantly lower VAS pain score with improved functional outcomes [63]. Similar study by Thahir et al. found inconclusive evidence concerning the use of PRP during meniscal repair and their impact on pain relief, functional recovery, rate of retear, and revision surgery [64]. These discrepancies may arise from differences in study design, sample sizes, PRP preparation methods, and outcome measures. Sakti et al. included studies with varying PRP preparation protocols, which could influence the concentration and efficacy of growth factors, whereas Thahir et al. focused on randomized controlled trials, potentially providing more robust evidence but with a narrower inclusion criterion. In addition, the definition of “failure” varied between studies, with some considering reoperation rates and others assessing functional outcomes. Hence, in view of conflicting results, further studies and RCTs are needed to better investigate the impact of PRP augmentation on meniscal repair long-term outcomes.

### 2.7. Rehabilitation

Rehabilitation protocols following meniscal repair or meniscectomy are critical for ensuring optimal recovery, preventing complications, and restoring knee function. The role of postoperative rehabilitation for meniscal tears has been evolving, but the debate around ideal protocols continues. The protocols must be tailored to the type of meniscal tear, the surgical procedure performed and personalized as per the patient's specific needs. In Phase 1 (0–6 weeks) following meniscal repair, postoperative rehabilitation focuses on protecting the healing meniscus with nonweight-bearing for 2–4 weeks, followed by partial weight-bearing and gentle range of motion exercises, aiming for 0°–90° knee flexion. In Phase 2 (6–12 weeks), full weight-bearing is typically allowed, with progressive strengthening exercises, closed-chain activities, and the introduction of low-impact activities such as cycling and swimming, while avoiding high-impact sports until 12–16 weeks [65, 66].

Following meniscectomy, rehabilitation is generally faster than after meniscal repair, but careful management is essential to avoid overloading the remaining meniscus. In Phase 1 (0–4 weeks), partial weight-bearing progresses to full weight-bearing by 4 weeks, with a focus on a gentle range of motion and quadriceps strengthening, while avoiding high-impact activities; in Phase 2 (4–12 weeks), full weight-bearing is allowed, and strengthening exercises such as leg presses and mini-squats are introduced, and low-impact activities such as cycling and swimming are permitted, with cautious return to running. Phase 3 (3–6 months) comprises dynamic exercise and plyometrics, and sport-specific drills such as jumping, cutting, and pivoting are integrated to restore full function and stability [67, 68].

### 2.8. Algorithm

To optimize outcomes in meniscal repair, surgeons should adopt a personalized approach that tailors repair techniques based on tear type, location, and patient factors. A practical algorithm for selecting repair techniques might involve the following:

- Tear type: Horizontal tears are treated with meniscal repair or partial meniscectomy if repair is not feasible, as they are more likely to heal well due to their alignment with the joint line. For vertical tears (longitudinal), repair is typically the first choice, especially if the tear is in the red–red or red–white zones, as these have better vascular supply. Complex or degenerative tears require hybrid approaches combining meniscal scaffold or biologic augmentation (e.g., PRP and stem cells), which may be necessary, especially in patients with poor healing potential, and when all options fail, meniscectomy is the best option.
- Tear location: Red–red zone tears are favorable for suturing or repair techniques, as this zone has adequate blood supply. Red–white zone tears require biologic augmentation or scaffold use for better healing outcomes. White–white zone tears are often not repairable, and partial meniscectomy is the preferred option, with biologic treatments being investigated for improving regeneration.
- Patient factors: Younger, more active patients may benefit more from meniscal repair or hybrid techniques, whereas older patients or those with lower activity levels may be more suited for meniscectomy or biologic augmentation to enhance recovery.
- Use of biologics: For challenging cases or patients with compromised healing potential, integrating biologics such as PRP, stem cells, or scaffolds to enhance tissue regeneration, especially in patients with complex tears or those in the red–white or white–white zones, is considered.

By following this algorithm, surgeons can choose the most appropriate repair technique for each patient, improving outcomes, recovery times, and long-term joint health.

### 2.9. Limitations

While the current body of literature on meniscal repair has contributed valuable insights, several limitations must be considered when interpreting the findings. First, the heterogeneity in study designs, with variations in surgical techniques, rehabilitation protocols, and patient populations, makes it difficult to draw definitive conclusions across studies. Second, many studies are of small sample sizes, particularly trials evaluating the use of PRP and other biologics, which limits the statistical power and generalizability of the results. Third, the lack of long-term follow-up data is another significant limitation, as it is unclear whether short-term improvements in outcomes translate into sustained long-term benefits. Fourth, the absence of standardized outcome measures across studies can make comparisons between different repair techniques and interventions challenging. Future research should prioritize large, well-designed randomized controlled trials comparing various adjunct biologics (e.g., PRP and stem cells) with conventional repair methods, as well as long-term studies assessing repair sustainability, failure rates, and their impact on joint health. In addition, patient-reported outcomes, biomechanical studies, and personalized treatment approaches based on tear type and location are crucial for improving recovery and treatment strategies.

The learning curve associated with repair techniques can affect their reproducibility, with less experienced surgeons potentially facing challenges in technical execution, biological integration, and managing complications; however, with increased experience, better training, and more standardized approaches, reproducibility and overall surgical outcomes are likely to improve over time through education, mentorship, and standardized protocols for hybrid techniques. Furthermore, ethical challenges in biologic therapies for meniscal repair, such as stem cell sourcing and allograft transplantation, include concerns over informed consent, the potential exploitation of vulnerable patients, and ensuring rigorous regulation of treatments with limited evidence of efficacy. In addition, issues such as donor consent, the risk of disease transmission with allografts, and equitable access to grafts raise further ethical considerations. Finally, cost-effectiveness analyses of biologics and advanced repair technologies are needed to ensure accessibility and affordability of these treatments in clinical practice.

## 3. Conclusion

Advancements in medical technology aim at preserving and repairing the meniscus while drifting away from partial and complete meniscectomy even among aged populations. Many techniques are being studied, and the current literature is reinforcing the importance of meniscal tear repair and salvage therapies for irreparable chronic and complex meniscal tears instead of meniscectomy. There exist some contraindications for meniscal tear repair, and rehabilitation is critical to ensure proper meniscal healing and return to full functional capacity. More research is needed with high evidence comparative studies and trials comparing different methods of meniscal repair for better individualization of treatment and optimizing functional outcomes.

## Data Availability

Data sharing is not applicable to this article as no datasets were generated or analyzed during the current study.

## References

[1] Mordecai S. C., Al-Hadithy N., Ware H. E., Gupte C. M. (2014). Treatment of Meniscal Tears: An Evidence Based Approach. *World Journal of Orthopedics*.

[2] Fairbank T. J. (1948). Knee Joint Changes After Meniscectomy. *The Journal of Bone and Joint Surgery. British Volume*.

[3] Barron D. W., Gill V. S., Boddu S. P., Beckett N. C., Tummala S. V., Chhabra A. (2025). Low Income, Younger Age, Female Sex, and Poor Mental Health Are Risk Factors for Diminished Access to Care Among Patients With Meniscus Tears. *Arthroscopy, Sports Medicine, and Rehabilitation*.

[4] Makris E. A., Hadidi P., Athanasiou K. A. (2011). The Knee Meniscus: Structure Function, Pathophysiology, Current Repair Techniques, and Prospects for Regeneration. *Biomaterials*.

[5] Li Z., Weng X. (2022). Platelet-Rich Plasma Use in Meniscus Repair Treatment: A Systematic Review and Meta-Analysis of Clinical Studies. *Journal of Orthopaedic Surgery and Research*.

[6] Bouji N., Wen S., Dietz M. J. (2022). Intravenous Antibiotic Duration in the Treatment of Prosthetic Joint Infection: Systematic Review and Meta-Analysis. *Journal of Bone and Joint Infection*.

[7] Liberati A., Altman D. G., Tetzlaff J. (2009). The PRISMA Statement for Reporting Systematic Reviews and Metaanalyses of Studies That Evaluate Health Care Interventions: Explanation and Elaboration. *Journal of Clinical Epidemiology*.

[8] Zhao Y., Zhu S., Wan Q. (2022). Understanding How and by Whom COVID-19 Misinformation is Spread on Social Media: Coding and Network Analyses. *Journal of Medical Internet Research*.

[9] (2024). Home-Clinicaltrials.Gov. https://clinicaltrials.gov/.

[10] Sayegh E. T., Matzkin E. (2022). Classifications in Brief: The International Society of Arthroscopy, Knee Surgery, and Orthopaedic Sports Medicine Classification of Meniscal Tears. *Clinical Orthopaedics and Related Research*.

[11] Yan W., Dai W., Cheng J. (2021). Histologically Confirmed Recellularization is a Key Factor That Affects Meniscal Healing in Immature and Mature Meniscal Tears. *Frontiers in Cell and Developmental Biology*.

[12] Klimkiewicz J. J., Shaffer B. (2002). Meniscal Surgery 2002 Update: Indications and Techniques for Resection, Repair, Regeneration, and Replacement. *Arthroscopy: The Journal of Arthroscopic & Related Surgery*.

[13] Matava M. J., Eck K., Totty W., Wright R. W., Shively R. A. (1999). Magnetic Resonance Imaging as a Tool to Predict Meniscal Reparability. *The American Journal of Sports Medicine*.

[14] Wu I. T., Hevesi M., Desai V. S. (2018). Comparative Outcomes of Radial and Bucket-Handle Meniscal Tear Repair: A Propensity-Matched Analysis. *The American Journal of Sports Medicine*.

[15] DeFroda S. F., Yang D. S., Donnelly J. C., Bokshan S. L., Owens B. D., Daniels A. H. (2020). Trends in the Surgical Treatment of Meniscal Tears in Patients With and Without Concurrent Anterior Cruciate Ligament Tears. *The Physician and Sportsmedicine*.

[16] Beaufils P., Pujol N. (2018). Meniscal Repair: Technique. *Orthopedie Traumatologie: Surgery & Research*.

[17] Krych A. J., LaPrade M. D., Cook C. S. (2020). Lateral Meniscal Oblique Radial Tears Are Common With ACL Injury: A Classification System Based on Arthroscopic Tear Patterns in 600 Consecutive Patients. *Orthopaedic Journal of Sports Medicine*.

[18] Golz A. G., Mandelbaum B., Pace J. L. (2022). All-Inside Meniscus Repair. *Current Reviews in Musculoskeletal Medicine*.

[19] Ozeki N., Seil R., Krych A. J., Koga H. (2021). Surgical Treatment of Complex Meniscus Tear and Disease: State of the Art. *Journal of ISAKOS*.

[20] Arnoczky S. P., Warren R. F. (1982). Microvasculature of the Human Meniscus. *The American Journal of Sports Medicine*.

[21] Fithian D. C., Kelly M. A. (2002). Meniscal Function and Biomechanics: Implications for Meniscal Repair. *Sports Medicine and Arthroscopy Review*.

[22] Bansal S., Keah N. M., Neuwirth A. L. (2017). Large Animal Models of Meniscus Repair and Regeneration: A Systematic Review of the State of the Field. *Tissue Engineering Part C Methods*.

[23] Cabaud H. E., Rodkey W. G., Fitzwater J. E. (1981). Medial Meniscus Repairs: An Experimental and Morphologic Study. *The American Journal of Sports Medicine*.

[24] Karia M., Ghaly Y., Al-Hadithy N., Mordecai S., Gupte C. (2019). Current Concepts in the Techniques, Indications and Outcomes of Meniscal Repairs. *European Journal of Orthopaedic Surgery and Traumatology*.

[25] Fox J. M., Rintz K. G., Ferkel R. D. (1993). Trephination of Incomplete Meniscal Tears. *Arthroscopy: The Journal of Arthroscopic & Related Surgery*.

[26] Zhang Z., Arnold J. A., Williams T., McCann B. (1995). Repairs by Trephination and Suturing of Longitudinal Injuries in the Avascular Area of the Meniscus in Goats. *The American Journal of Sports Medicine*.

[27] Cannon W. D., Vittori J. M. (1992). The Incidence of Healing in Arthroscopic Meniscal Repairs in Anterior Cruciate Ligament-Reconstructed Knees Versus Stable Knees. *The American Journal of Sports Medicine*.

[28] Everhart J. S., Higgins J. D., Poland S. G., Abouljoud M. M., Flanigan D. C. (2018). Meniscal Repair in Patients Age 40 Years and Older: A Systematic Review of 11 Studies and 148 Patients. *The Knee*.

[29] Sherman S. L., Albersheim M. (2025). Editorial Commentary: Older Age is Not a Contraindication to Meniscal Repair. *Arthroscopy: The Journal of Arthroscopic & Related Surgery*.

[30] Ogawa H., Matsumoto K., Sengoku M., Yoshioka H., Akiyama H. (2020). Arthroscopic Repair of Horizontal Cleavage Meniscus Tears Provides Good Clinical Outcomes in Spite of Poor Meniscus Healing. *Knee Surgery, Sports Traumatology, Arthroscopy*.

[31] Joshi A., Basukala B., Singh N., Hama B., Bista R., Pradhan I. (2020). Outside-In Repair of Longitudinal Tear of Medial Meniscus: Suture Shuttle Technique. *Arthroscopy Techniques*.

[32] Wright R. W., Huston L. J., Haas A. K. (2023). Ten-Year Outcomes of Secondgeneration, All-Inside Meniscal Repair in the Setting of ACL Reconstruction. *Journal of Bone and Joint Surgery*.

[33] Gill S., Diduch D. (2002). Outcomes After Meniscal Repair Using the Meniscus Arrow in Knees Undergoing Concurrent Anterior Cruciate Ligament Reconstruction. *Arthroscopy: The Journal of Arthroscopic & Related Surgery*.

[34] Arnoczky S. P., Lavagnino M. (2001). Tensile Fixation Strengths of Absorbable Meniscal Repair Devices as a Function of Hydrolysis Time: An In Vitro Experimental Study. *The American Journal of Sports Medicine*.

[35] Masoudi A., Beamer B. S., Harlow E. R. (2015). Biomechanical Evaluation of an all-Inside Suture-Based Device for Repairing Longitudinal Meniscal Tears. *Arthroscopy: The Journal of Arthroscopic & Related Surgery*.

[36] Popescu D., Sastre S., Caballero M. (2010). Meniscal Repair Using the Fast-Fix Device in Patients With Chronic Meniscal Lesions. *Knee Surgery, Sports Traumatology, Arthroscopy*.

[37] Boksh K., Shepherd D. E. T., Espino D. M., Ghosh A., Boutefnouchet T., Aujla R. (2024). Suture Tapes Show Superior Biomechanical Properties and Greater Meniscal Healing Compared to Conventional Sutures in Posterior Meniscal Root Tear Repairs: A Systematic Review. *Knee Surgery, Sports Traumatology, Arthroscopy*.

[38] Buckland D., Sadoghi P., Wimmer M. D. (2015). Meta-Analysis on Biomechanical Properties of Meniscus Repairs: Are Devices Better Than Sutures?. *Knee Surgery, Sports Traumatology, Arthroscopy*.

[39] Rothermel S. D., Smuin D., Dhawan A. (2018). Are Outcomes After Meniscal Repair Age Dependent? A Systematic Review. *Arthroscopy: The Journal of Arthroscopic & Related Surgery*.

[40] Kocabey Y., Chang H. C., Brand J. C., Nawab A., Nyland J., Caborn D. N. (2006). A Biomechanical Comparison of the Fast-Fix Meniscal Repair Suture System and the Rapidloc Device in Cadaver Meniscus. *Arthroscopy: The Journal of Arthroscopic & Related Surgery*.

[41] Sherman S. L., Askew N., Nherera L. M., Searle R. J., Flanigan D. C. (2024). An All-Suture-Based Technique for Meniscal Repair is Cost-Effective in Comparison to Partial Meniscectomy for Horizontal Cleavage Tears. *Arthroscopy, Sports Medicine, and Rehabilitation*.

[42] Barber F. A., Schroeder F. A., Oro F. B., Beavis R. C. (2008). FasT-Fix Meniscal Repair: Mid-Term Results. *Arthroscopy: The Journal of Arthroscopic & Related Surgery*.

[43] Tachibana Y., Sakaguchi K., Goto T., Oda H., Yamazaki K., Iida S. (2010). Repair Integrity Evaluated by Second-Look Arthroscopy After Arthroscopic Meniscal Repair With the FasT-Fix During Anterior Cruciate Ligament Reconstruction. *The American Journal of Sports Medicine*.

[44] Dzidzishvili L., Jackson G. R., Allende F., Mameri E. S., Allahabadi S., Chahla J. (2025). Meniscal Radial Tears Repaired With All-Inside and Inside-Out Techniques Result in Improved Clinical Outcome Scores, But Inside-Out Repairs May Be Associated With Higher Failure Rates Clinically and on Second-Look Arthroscopy: A Systematic Review. *Arthroscopy: The Journal of Arthroscopic & Related Surgery*.

[45] Bian Y., Wang H., Zhao X., Weng X. (2022). Meniscus Repair: Up-to-Date Advances in Stem Cell-Based Therapy. *Stem Cell Research & Therapy*.

[46] Berton A., Longo U. G., Candela V. (2020). Quantitative Evaluation of Meniscal Healing Process of Degenerative Meniscus Lesions Treated With Hyaluronic Acid: A Clinical and MRI Study. *Journal of Clinical Medicine*.

[47] Tramś E., Kamiński R. (2024). Molecular Biology of Meniscal Healing: A Narrative Review. *International Journal of Molecular Sciences*.

[48] Ding G., Du J., Hu X., Ao Y. (2022). Mesenchymal Stem Cells From Different Sources in Meniscus Repair and Regeneration. *Frontiers in Bioengineering and Biotechnology*.

[49] Mazy D., Wang J., Dodin P., Lu D., Moldovan F., Nault M. L. (2024). Emerging Biologic Augmentation Strategies for Meniscal Repair: A Systematic Review. *BMC Musculoskeletal Disorders*.

[50] Utrilla G. S., Degano I. R., D’Ambrosi R. (2024). Efficacy of Platelet-Rich Plasma in Meniscal Repair Surgery: A Systematic Review of Randomized Controlled Trials. *Journal of Orthopaedics and Traumatology*.

[51] Alentorn-Geli E., Cuscó X., Navarro J. (2020). Circumferential-Surrounding (“Sandwich”) Meniscal Repair: A Salvage Technique to Save the Meniscus. *Arthrosc Tech*.

[52] Smith & Nephew (2025). New Data Shows NOVOSTITCH Systems Achieve 82% Success Rate in Challenging Horizontal Cleavage Meniscal Tears. https://www.smith-nephew.com/news/2020/11/17/20201117-new-data-shows-sns-novostitch-systems-achieve-82-success-rate?utm.

[53] Henning C. E. (1990). Current Status of Meniscus Salvage. *Clinics in Sports Medicine*.

[54] Beatrice Tan J. N., James Loh S. Y. (2021). An Approach to Chronic and Displaced Bucket Handle Meniscal Tear—Assessment, Repair (Push-and-Pull Technique), or Salvage. *Arthroscopy Techniques*.

[55] Zaffagnini S., Grassi A., Macchiarola L. (2019). Meniscal Allograft Transplantation is an Effective Treatment in Patients Older Than 50 Years But Yields Inferior Results Compared With Younger Patients: A Case-Control Study. *Arthroscopy: The Journal of Arthroscopic & Related Surgery*.

[56] Yale Medicine (2025). *Meniscal Allograft Transplantation*.

[57] Vasta S., Zampogna B., Hartog T. D., El Bitar Y., Uribe-Echevarria B., Amendola A. (2022). Outcomes, Complications, and Reoperations After Meniscal Allograft Transplantation. *Orthopaedic Journal of Sports Medicine*.

[58] Wasyłeczko M., Sikorska W., Chwojnowski A. (2020). Review of Synthetic and Hybrid Scaffolds in Cartilage Tissue Engineering. *Membranes*.

[59] Bulgheroni E., Grassi A., Campagnolo M., Bulgheroni P., Mudhigere A., Gobbi A. (2016). Comparative Study of Collagen Versus Synthetic-Based Meniscal Scaffolds in Treating Meniscal Deficiency in Young Active Population. *Cartilage*.

[60] Reale D., Lucidi G. A., Grassi A., Poggi A., Filardo G., Zaffagnini S. (2022). A Comparison Between Polyurethane and Collagen Meniscal Scaffold for Partial Meniscal Defects: Similar Positive Clinical Results at a Mean of 10 Years of Follow-Up. *Arthroscopy: The Journal of Arthroscopic & Related Surgery*.

[61] Bhise M. G., Patel L., Patel K. (2024). 3D Printed Medical Devices: Regulatory Standards and Technological Advancements in the USA, Canada and Singapore-A Cross-National Study. *International Journal of Pharmaceutical Investigation*.

[62] Stocco E., Porzionato A., De Rose E., Barbon S., De Caro R., Macchi V. (2022). Meniscus Regeneration by 3D Printing Technologies: Current Advances and Future Perspectives. *Journal of Tissue Engineering*.

[63] Sakti M., Paturusi I. A., Singjie L. C., Kusuma S. A. (2024). The Use of Platelet-Rich Plasma Augmentation in Meniscus Repair Results in a Lower Failure Rate Than in the Control Group: A Systematic Review From Meta-Analysis. *Arthroscopy, Sports Medicine, and Rehabilitation*.

[64] Thahir M., Misbah I., Bhaskaran J., Syed N. H., Ashraf M., Balasubramanian N. (2024). Efficacy of Intraoperative Platelet-Rich Plasma After Meniscal Repair: Systematic Review and Meta-Analysis. *Indian Journal of Orthopaedics*.

[65] Massachusetts General Hospital (2025). Rehabilitation Protocol for Meniscus Repair. https://www.massgeneral.org/assets/mgh/pdf/orthopaedics/sports-medicine/physical-therapy/rehabilitation-protocol-for-meniscus-repair.pdf.

[66] VCU Health (2025). Meniscal Repair Rehabilitation Protocol. https://www.vcuhealth.org/media/vcuhealth/media/media/file/meniscal-repair-protocol.pdf.

[67] Brigham and Women’s Hospital (2025). Partial Meniscectomy or Debridement Rehabilitation Protocol. https://www.brighamandwomens.org/assets/bwh/patients-and-families/pdfs/knee--partial-meniscectomy-or-debridement.pdf.

[68] Massachusetts General Hospital (2025). Rehabilitation Protocol for Partial Meniscectomy. https://www.massgeneral.org/assets/mgh/pdf/orthopaedics/sports-medicine/physical-therapy/rehabilitation-protocol-for-meniscectomy.pdf.

